# P-114. Efficacy of Vaginal-Spraying *Bacillus* Spore Probiotics in Supportive Treatment of Sexual Transmission Infections: A Randomized, Double-Blind, Placebo-Controlled Clinical Trial

**DOI:** 10.1093/ofid/ofae631.321

**Published:** 2025-01-29

**Authors:** Anh Thi Van Nguyen, Huyen Thi Bui, Hong Thi Ngo, Anh Thi Phuong Bui, Huyen Thi Nguyen, Van Cam Tran, Anh Hoa Nguyen

**Affiliations:** ANABIO R&D Ltd., Hanoi, Ha Noi, Vietnam; ANABIO R&D Ltd., Hanoi, Ha Noi, Vietnam; Bac Ninh Center for Disease Control, Bac Ninh, Bac Ninh, Vietnam; ANABIO R&D Ltd., Hanoi, Ha Noi, Vietnam; LiveSpo Pharma Ltd., Hanoi, Ha Noi, Vietnam; National Hospital of Dermatology and Venereology, Hanoi, Ha Noi, Vietnam; LiveSpo Pharma Ltd., Hanoi, Ha Noi, Vietnam

## Abstract

**Background:**

Sexually Transmitted Infections (STIs) are common gynecological infections worldwide. While antibiotics drugs are typically utilized to treat STIs caused by pathogenic bacteria, the over use of antibiotics may lead to side effects and the emergence of drug resistance. Hence, vaginal-spraying probiotics could offer a safe and effective symptomatic treatment for STIs, presenting a promising alternative to standard therapy.

The Effect of Vaginal-Spraying Bacillus Spore Probiotics (LiveSpo X-SECRET) in Reducing Gardenella vaginlis Concentrations in Vaginal Swab.

**Figure:**
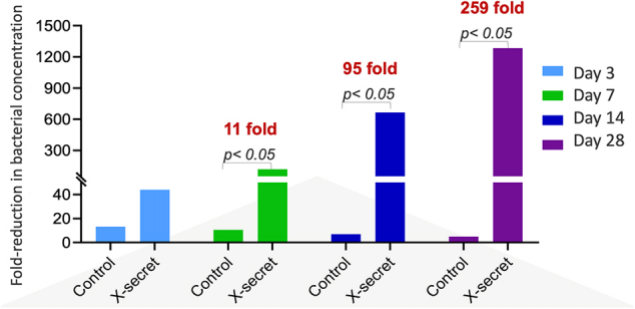

**Methods:**

We conducted a randomized, double-blind, placebo-controlled clinical trial to evaluate the efficacy of vaginal-spraying probiotics containing spores of *Bacillus subtilis*, *B. clausii*, and *B. coagulans* (> 1 billion/mL) in supporting the treatment of vaginal infections. The study comprised 100 eligible patients visiting Bac Ninh Center for Disease Control, randomly divided into 2 groups (n = 50/group). The standard treatment regimen lasted 7 days and was maintained for 28 days. The Control group received NaCl 0.9% physiological saline in addition, while the X-Secret group was additionally treated with LiveSpo X-SECRET. This trial was registered at clinicaltrials.gov as NCT06165354.

**Results:**

LiveSpo X-SECRET did not cause skin or vaginal mucosal irritation and exhibited supportive effects in significantly reducing typical symptoms of vaginal infection, indicated by a lower percentage of patients experiencing vaginal odor, vaginal itching, urinary pain, and abnormal vaginal discharge coloration in the X-Secret group observed at days 3, 7, 14, and 28, compared to the Control group. The clinical findings were supported by real-time PCR detection of STIs, indicating a reduction in pathogenic *G. vaginalis* concentration by approximately 11-260 folds and an increase in beneficial *Lactobacillus* sp. by around 10^2^-10^3^ folds, more effectively observed in vaginal swabs from the X-Secret group compared to the Control group on days 3, 7, 14, and 28. Additionally, 16S rRNA metagenome analysis data revealed that LiveSpo X-SECRET restored vaginal microbiota by increasing the density of Lactobacillus while reducing some harmful genera.

**Conclusion:**

Vaginal-spraying *Bacillus* spore probiotics (LiveSpo X-SECRET) can be considered a simple and effective agent for supportive treatment of sexually transmitted infections.

.

**Disclosures:**

**Huyen Thi Nguyen, MSc. in Biotechnology**, LiveSpo Pharma Ltd.: Expert Testimony|LiveSpo Pharma Ltd.: Huyen Thi Nguyen receives a monthly salary from LiveSpo Pharma., and does not receive any additional compensation for her involvement in this study. **Anh Hoa Nguyen, PhD. in Life Sciences**, LiveSpo Pharma Ltd.: Board Member|LiveSpo Pharma Ltd.: Anh Hoa Nguyen receives a monthly salary from LiveSpo Pharma , and does not receive any additional compensation for his involvement in this study.|LiveSpo Pharma Ltd.: Ownership Interest

